# Trophic Activity of Human P2X7 Receptor Isoforms A and B in Osteosarcoma

**DOI:** 10.1371/journal.pone.0107224

**Published:** 2014-09-16

**Authors:** Anna Lisa Giuliani, Davide Colognesi, Tiziana Ricco, Carlotta Roncato, Marina Capece, Francesca Amoroso, Qi Guang Wang, Elena De Marchi, Allison Gartland, Francesco Di Virgilio, Elena Adinolfi

**Affiliations:** 1 Department of Morphology, Surgery and Experimental Medicine, Section of Experimental Pathology, Oncology and Biology, University of Ferrara, Ferrara, Italy; 2 Centre For Tissue Injury and Repair, The University of Manchester, Manchester, United Kingdom; 3 The Mellanby Center for Bone Research, the University of Sheffield, Sheffield, United Kingdom; University Paris Sud, France

## Abstract

The P2X7 receptor (P2X7R) is attracting increasing attention for its involvement in cancer. Several recent studies have shown a crucial role of P2X7R in tumour cell growth, angiogenesis and invasiveness. In this study, we investigated the role of the two known human P2X7R functional splice variants, the full length P2X7RA and the truncated P2X7RB, in osteosarcoma cell growth. Immunohistochemical analysis of a tissue array of human osteosarcomas showed that forty-four, of a total fifty-four tumours (81.4%), stained positive for both P2X7RA and B, thirty-one (57.4%) were positive using an anti-P2X7RA antibody, whereas fifteen of the total number (27.7%) expressed only P2X7RB. P2X7RB positive tumours showed increased cell density, at the expense of extracellular matrix. The human osteosarcoma cell line Te85, which lacks endogenous P2X7R expression, was stably transfected with either P2X7RA, P2X7RB, or both. Receptor expression was a powerful stimulus for cell growth, the most efficient growth-promoting isoform being P2X7RB alone. Growth stimulation was matched by increased Ca^2+^ mobilization and enhanced NFATc1 activity. Te85 P2X7RA+B cells presented pore formation as well as spontaneous extracellular ATP release. The ATP release was sustained in all clones by P2X7R agonist (BzATP) and reduced following P2X7R antagonist (A740003) application. BzATP also increased cell growth and activated NFATc1 levels. On the other hand cyclosporin A (CSA) affected both NFATc1 activation and cell growth, definitively linking P2X7R stimulation to NFATc1 and cell proliferation. All transfected clones also showed reduced RANK-L expression, and an overall decreased RANK-L/OPG ratio. Mineralization was increased in Te85 P2X7RA+B cells while it was significantly diminished in Te85 P2X7RB clones, in agreement with immunohistochemical results. In summary, our data show that the majority of human osteosarcomas express P2X7RA and B and suggest that expression of either isoform is differently coupled to cell growth or activity.

## Introduction

Osteosarcoma is the most common primary bone cancer, accounting for approximately six percent of all new paediatric tumours per year [1], [2]. Histology of cancer lesions shows a mixture of proliferating osteoblast cells, responsible for sclerosis, and activated osteoclasts, responsible for bone resorption and osteolysis. To date, few treatments to counteract pathologic bone remodelling and alleviate the associated pain are available [1]. Among these the monoclonal antibody denosumab, which blocks receptor activator of nuclear factor kB ligand (RANK-L), is giving promising clinical results for treatment of cancer related bone disorders [3]. The RANK-RANKL system is the main activator of osteoclast formation and function. Osteoblast can express or secrete either RANKL or its antagonist osteoprotegerin (OPG) to induce osteoclasts mediated resorption or to stop it, respectively. Interestingly, RANK-L levels have been suggested to be reduced in advanced stage osteosarcoma [4].

Recent *in vitro* and *in vivo* evidence show that the P2X7 receptor (P2X7R) has a central role in carcinogenesis enhancing tumour cell growth [5], [6], tumour-associated angiogenesis [5] and cancer invasiveness [7], [8]. These data further support previous *in vitro* reports demonstrating that P2X7R expression increases cell proliferation [9], [10], mitochondria and endoplasmic reticulum Ca^2+^ levels [10], [11], vascular endothelial growth factor (VEGF) secretion [12], and agarose infiltration [13]. Furthermore, a growing literature confirms early findings documenting an increased P2X7R expression in human tumours (recently reviewed in [14], [15]). Although P2X7R is known to modulate osteoblast proliferation and osteodeposition [16], no direct proof of P2X7R involvement in bone cancers was available.

The P2X7R is an ATP-gated ion channel that, upon sustained stimulation with millimolar ATP concentrations, drives opening of a non-selective large conductance pore that admits hydrophilic molecules of MW up to 900 Da. Besides its natural ligand ATP, the most potent, albeit non strictly selective, pharmacological agonist is 2′,3′-(4-benzoyl)-benzoyl-ATP (BzATP). Several single nucleotide polymorphisms (SNPs), either loss- or gain-of-function, are known, some of them associated to diseases as different as familiar chronic lymphocytic leukaemia, bipolar-disorders or osteoporosis (recently reviewed in [17]). Moreover, nine different naturally occurring human P2X7R splice variants (indicated as P2X7RA to J) have been identified, P2X7RA being the well-characterized full-length receptor [18], [19]. Four out of the nine splice variants, P2X7RB, P2X7RE, P2X7RG and P2X7RJ, lack the extended C-terminal tail typical of P2X7RA. Among these, P2X7RJ acts as dominant negative [19], while P2X7RB, unique among truncated P2X7R splice variants, is a functional ion channel, although unable to form the large conductance pore [18]. P2X7RB retains an intron between exons 10 and 11, causing the addition of 18 extra aminoacids after residue 346 followed by a stop codon. These modifications do not affect receptor pharmacology as P2X7RA and P2X7RB share the same agonists and antagonists profile [18].

We recently investigated the effect of P2X7RB expression in HEK293 cells, showing that, besides P2X7RA, also this isoform exerts a trophic activity [13]. Co-expression of both P2RX7A and B, which are expressed in many different human tissues, further potentiated cell growth. Moreover, P2X7RA and B co-associated on the plasma membrane increasing large conductance pore opening, endoplasmic reticulum Ca^2+^ levels and NFATc1 activity [13].

P2X7R is expressed in primary human osteoblasts, as well as in primary rat osteoblasts and human osteosarcoma cell lines [20]–[22]. Furthermore, data from *p2rx7^−/−^* mice show that lack of P2X7R is associated to reduction of periosteal bone formation [23] and osteogenic response to mechanical loading [24]. These data are also corroborated by recent findings linking P2X7R SNPs to osteoporosis [25]. Several P2X7R loss-of-function SNPs are indeed associated to increased osteoporosis risk [26]–[28], while gain-of-function variants are protective [29], [30]. P2X7R is expressed both in osteoblasts [20] and osteoclasts [31]–[33] and might play a central role in osteoblasts-osteoclasts crosstalk via calcium oscillations [34] and other signalling pathways [35]. P2X7R promotes osteogenesis by stimulating osteoblast proliferation as well as osteodeposition [16] through a series of different pathways including c-*fos* [36], ERK [37], PI3K [38] and COX [16]. Finally, P2X7R likely mediates osteoblast ATP release, as P2X7R blockers inhibit ATP secretion [39], [40].

Aim of our study was to shed light on the role of P2X7R in human osteosarcoma and to reveal possible different effects of the two isoforms A and B of the receptor. P2X7RA and B expression was investigated in human osteosarcoma tissue arrays, whereas their function was assessed by transfection into the human osteosarcoma Te85 cell line. Our data reveal that P2X7R isoforms are widely expressed in human osteosarcomas, and that they differently modulate cell proliferation and mineralization.

## Materials and Methods

### Reagents and antibodies

Whole cell culture reagents, lipofectamine LTX, RNA extraction PureLink Kit and Fura 2 AM were purchased from Life technologies (Milan, Italy). Taq Man Reverse transcription kit was from Roche (Milan, Italy), whereas anti P2X7 polyclonal antibody (also referred as anti-P2X7R-Cter), BzATP (2′,3′-(4-benzoyl)-benzoyl-ATP), cell dissociation solution, bovine serum albumin (BSA), ethidium bromide, sulfinpyrazone, hygromycin B, cyclosporin A (CSA), apyrase, Mayer's haematoxylin, dexamethasone, ascorbic acid and alizarin red S were from Sigma-Aldrich (Milan, Italy). Anti-P2XR7 monoclonal antibody (mAb), targeting the extracellular loop of P2X7R [41] (also referred as anti-P2X7R-ec), was kindly provided by Professor James Wiley (Florey Neuroscience Institutes, University of Melbourne, Australia). Secondary, HRP-conjugated, goat anti-rabbit and goat anti-mouse antibodies, and Liquid diaminobenzidine (DAB) Substrate Chromogen System were from Dako (Milan, Italy). Anti-Ki67 rabbit polyclonal antibody and NBT/BCIP plus suppressor were from Thermo Scientific (Rockford, IL, USA). Alkaline phosphatase (AP)-conjugated goat anti-rabbit antibody was from Bethyl (Montgomery, TX, USA). Access RT-PCR system kit and ENLITEN rLuciferase/Luciferin reagent were purchased from Promega (Milan, Italy). G418 sulphate was from Calbiochem (La Jolla, CA, USA). A740003 was from Tocris (Ellisville, MO, USA).

### Immunohistochemistry and tumour specimens cell counts

A panel of 54 different osteosarcoma specimens was analysed using tissue array slides CV2 human, osteosarcoma Super Biochips (Super Biochips, South Korea distributed by Tema Ricerca, Bologna, Italy). Human samples were collected by Super Biochips according to Korean and United States laws (Code of federal regulation 45CFR 46.101(b)) ensuring that rights, welfare and privacy of human subjects were protected. An External Review Board, composed of pathologists from major hospitals and legal experts, also reviewed procedures. Slides were heated at 60°C for 20 min, deparaffinized with xylene and rehydrated by sequential passages through decreasing concentrations of ethanol. Endogenous peroxidase activity was blocked by 15 min incubation at room temperature with 0.3% H_2_O_2_ in TBS (50 mM Tris, 150 mM NaCl, pH 7,6). After three rinses in wash buffer (0.025% Triton X-100 in TBS), tissue sections were maintained for 2 hours at room temperature with a blocking solution (1% BSA, 10% FBS in TBS), and incubated overnight at 4°C with either a mouse anti-P2X7R mAb (anti-P2X7R-ec) (65 µg/ml) or a rabbit polyclonal anti-P2X7R antibody (anti-P2X7R-Cter) (20 µg/ml) in incubation solution (1% BSA in TBS). Sections were rinsed twice in wash buffer, incubated for 60 min at room temperature with appropriated HRP conjugated secondary antibodies (goat anti-mouse and goat anti-rabbit, respectively) diluted 1∶100 in incubation solution, washed in TBS, and submitted to 6–10 min incubation at room temperature with Liquid diaminobenzidine (DAB) Substrate Chromogen System (Dako). Counterstaining was performed with Mayer's haematoxylin. The same procedure was applied to 5 µm tissue sections from tumours obtained by sub-cutaneous inoculation of either HEK293-mock or HEK293-P2X7RA cells into nude mice (see [5]), used as negative and positive controls, respectively. Double immunostaining was performed applying simultaneously the two anti-P2X7R antibodies, at the same conditions as above. Furthermore, co-immunostaining using the monoclonal anti-P2X7R-ec plus a polyclonal anti-Ki67 antibody (diluted 1∶10 in incubation solution) was carried out after antigen unmasking performed for 30 min at 160°C in citrate buffer, pH 6.0. In both cases, after washes, slides were incubated with a mix of the two secondary antibodies, i.e. an anti-mouse HRP conjugated and an anti-rabbit AP conjugated. Chromogens were finally applied sequentially: DAB first and NBT/BCIP plus suppressor subsequently. Counterstaining was carried out as above. Images were acquired with a Nikon Eclipse H550L microscope (Nikon, Firenze, Italy) using the NIS-Elements software (Nikon). Tumour cell numbers were evaluated by counting haematoxylin stained nuclei with Image J software (Image J1.47v Wayne Rasband, NIH, USA).

### Cell cultures and transfections

Human osteosarcoma Te85 cell line [42] was previously used in Gartland's laboratory [20]. Cells were grown in DMEM+Glutamax medium supplemented with 10% FBS, 100 U/ml penicillin and 100 mg/ml streptomycin (complete culture medium). Plasmids containing human P2X7RA and P2X7RB were previously obtained in our laboratory [10], [13] respectively by inserting P2X7RA sequence in pcDNA 3.1 vector, carrying gentamicin resistance, and P2X7RB sequence in pcDNA 3.1 Hygro, containing hygromicin B resistance. Te85 cells expressing P2X7RA (Te85-P2X7RA), P2X7RB (Te85-P2X7RB), both (Te85-P2X7RA+B) or the empty vectors (Te85-mock) were obtained by transfection with lipofectamin LTX and antibiotic selection with either G418 sulphate (0.8–0.2 mg/ml), hygromicyn (0.2–0.1 mg/ml) or both, as previously described [13]. Serial dilutions allowed obtaining single cell clones that were tested at fluorimeter for changes in cytosolic Ca^2+^ concentration and ethidium bromide up-take (see below). Among all clones evaluated, at least two for each cell type were employed for subsequent experiments. Where not reported data obtained with Te85-mock cells were not significantly different from Te85-wt controls.

### RNA extraction

Cells lysates were obtained with TRIZOL reagent (Life technologies), and RNA was extracted using PureLink RNA Mini Kit. RNA content was determined using a Nanodrop 2000 spectrophotometer (Thermo Scientific, Milano, Italia) and RNA integrity was checked by electrophoresis on 1.5% agarose gel in Tris Borate EDTA (TBE) (89 mM Tris, 89 mM boric acid, 20 mM EDTA, pH 8.0). Gels were analysed by a RED analyser (Cell Biosciences, Santa Clara, CA, USA).

### RT-PCR

Semi quantitative PCR was performed as previously described [13] with Promega RT-PCR access kit (Promega, Milan, Italy) using 200–500 ng RNA/sample as template. Primers used were:

Forward 5′ AGATGCTGGAGAATGGAGTG 3′, reverse 5′ TTCTCGTGGTGTAGTTGTGG 3′ for P2X7RA.

Forward 5′ CCCATCGAGGCAGTGGA 3′, Reverse 5′ TTCTCGTGGTGTAGTTGTGG 3′ for P2X7RB.

Forward 5′GAAAATGGAGCTCCTGGTCA 3′, Reverse 5′ACCATTGGCACCTTTAGCAC 3′, for collagen I.

Forward 5′ CCTCTGACTTCAACAGCCAC 3′, Reverse 5′ CATGACAAGGTGCGGCTCCC 3′ for G3PDH.

### Flow Cytometry

Two million cells were re-suspended in PBS with the addition of 200 µg of anti-P2X7R-ec (see reagents and antibodies) at a final concentration of 25 µg/ml. Secondary antibody was an anti-mouse FITC-conjugated rabbit polyclonal antibody diluted 1∶200. Fluorescence was measured using a BD FACScan flow cytometer (Becton Dickinson, Milan, Italy), data were analysed with BD cell quest software and expressed as Mean Fluorescence Intensity after subtraction of secondary antibody values.

### Measurement of intracellular calcium concentration ([Ca^2+^]_i_)

Changes in the cytosolic free Ca^2+^ concentration were measured in a thermostat controlled (37°C) and magnetically stirred Cary Eclipse Fluorescence Spectrophotometer (Agilent Technologies, Cernusco SN, Milan, Italy) with the fluorescent indicator fura-2/acetoxymethyl ester (fura-2/AM) as previously described [10]. Briefly, 2×10^6^ cells were loaded with 2 µM fura-2/AM for 30 min in the presence of 250 µM sulfinpyrazone in saline solution (125 mM NaCl, 5 mM KCl, 1 mM MgSO_4_, 1 mM NaH_2_PO_4_, 20 mM HEPES, 5.5 mM glucose, 5 mM NaHCO_3_, and 1 mM CaCl_2_, pH 7.4), rinsed, and re-suspended at a final concentration of 10^6^/ml in the same buffer. When required, cells were re-suspended in Ca^2+^-free saline. Excitation ratio and emission wavelength were 340/380 and 505 nm, respectively.

### Measurement of plasma membrane permeabilization

Increases in plasma membrane permeability were measured by monitoring the ethidium bromide up-take. Briefly, 5×10^5^ cells were re-suspended in Ca^2+^-free saline solution, incubated in a thermostat controlled (37°C) and magnetically stirred fluorimetric cuvette in the presence of 20 µM ethidium bromide and challenged with the agonist. Full permeabilization was obtained by adding digitonin (100 µM) at the end of the experiment. Fluorescence emission was measured at an excitation/emission wavelength couple of 360/580 nm.

### Extracellular ATP measurement

Extracellular ATP was measured in the culture supernatants with ENLITEN rLuciferase/Luciferin reagent, as per manufacturer's instructions. Briefly, 5×10^4^ cells per well were plated in 96 well plates. Following adhesion cells were incubated for 24 hours in the presence or absence of the required stimuli or inhibitors. Luminescence from cell supernatants was measured following addition of 100 µl of ENLITEN reagent per well, in a Victor2 Perkin Elmer luminometer (Perkin Elmer, Wellesley, MA, USA).

### Proliferation assay

For cell growth assay 10^5^ cells/ml were plated in 6-well plates in DMEM+Glutamax medium without FBS and maintained at 37°C in a CO_2_ incubator. Cells were counted at various time intervals (24, 48, 72 hours) in a Bürker chamber with a phase contrast Olympus microscope (Olympus Life Science Europe, Hamburg, Germany).

### NFATc1 activation assay

Nuclear extracts were obtained with the nuclear Extract Kit (Active Motif, Rixensart, Belgium). The NFATc1 activation assay was performed using a nuclear extract from PHA-stimulated Jurkat cells for comparison with the TransAM NFATc1 ELISA kit (Active Motif, Vinci, Biochem, Vinci, Italy) as per manufacturer's instructions.

### Real-time PCR

Expression of receptor activator of nuclear factor kappa B-ligand (RANK-L) and osteoprotegerin (OPG) was determined by real-time PCR in a Step One Real-Time PCR system (Applied Biosystems). Reverse transcription was performed starting from 1 µg of RNA/sample using the High capacity cDNA Reverse transcription kit (Applied Biosystems) following manufacturer's instructions. For real-time PCR, 2 µl of cDNA were used as template. Amplification was performed with predesigned Taqman probes (Applied Biosystem) for RANK-L, OPG and GAPDH as reference mRNA. A comparative CT experiment (ΔΔCT) was run to allow determination of the change of expression (fold increase) of the target cDNA in the test sample relative to Te85 wt reference sample.

### Mineralization

Cells were seeded at a density of 10^5^ cells per well of 48-well plates, and cultured in complete culture medium. Once confluent, cells were cultured for 14 days with osteogenic medium (DMEM+0.5% FCS +10 nM dexamethasone +50 µg/ml ascorbic acid), with medium change every 2–3 days. After 14 days, the osteogenic medium was supplemented with 5 mM inorganic phosphate and cells were cultured for further 7 days, with medium change every 2–3 days. On completion of the culture, the cells were washed twice with PBS and fixed with 100% ethanol for at least 1 hour at 4°C. After fixation, cells were washed Twice with PBS and incubated in 40 mM alizarin red S (pH 4.2) for 90 min on an orbital shaker. The excess, unbound stain was completely removed by washing cells with 95% ethanol. The plates were then left to air-dry overnight, before scanning in a flatbed scanner at 1200 dpi and images analysed using Image J software.

### Statistics

Unless otherwise stated, data shown are mean ± SE. Test of significance was performed with Student's t test using Graphpad InStat (GraphPad Software Inc, San Diego, CA, USA). Coding: *p<0.05, **p<0.01, ***p<0.001.

## Results

### Human osteosarcomas express P2X7RA and P2X7RB

**Figure 1:**
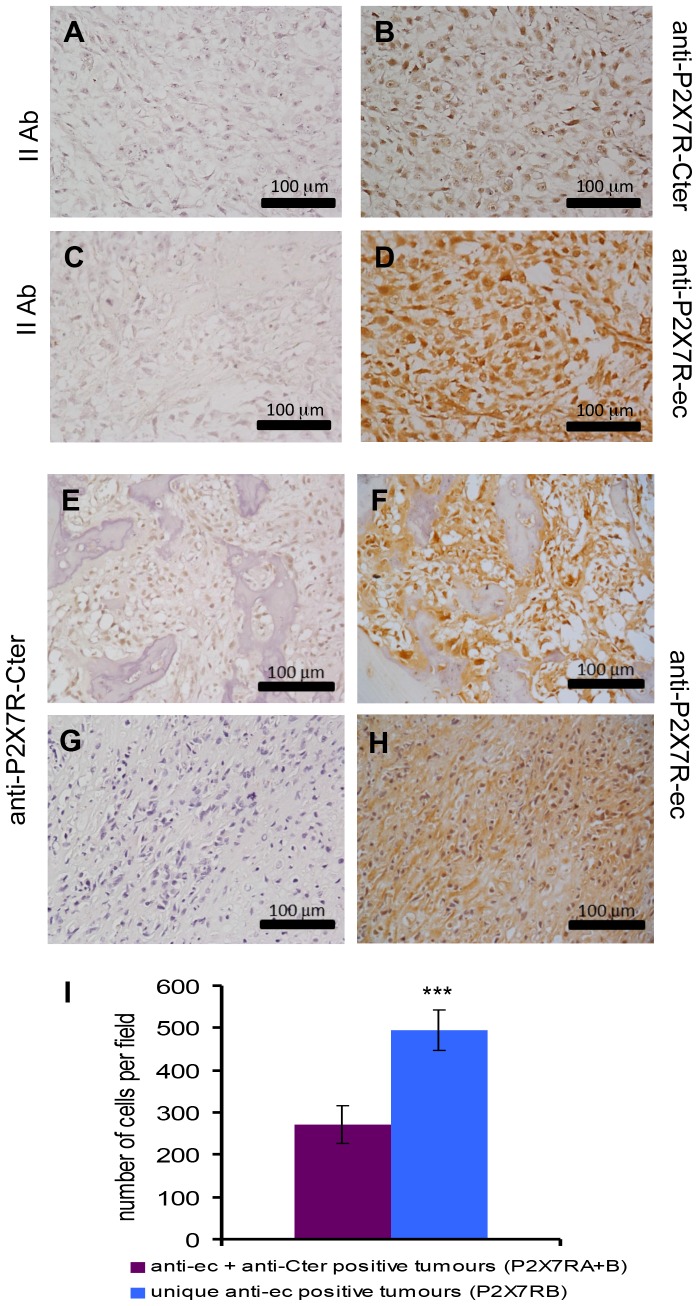
Human osteosarcomas express P2X7RA and P2X7RB. Paraffin embedded osteosarcoma tissue array was assayed by immonohistochemistry for P2X7R expression (see Materials and Methods) and representative samples are shown. (B): osteosarcoma stained with a polyclonal antibody recognizing P2X7R C-terminal domain (anti-P2X7R-Cter). (D): osteosarcoma stained with a monoclonal antibody recognizing P2X7R extracellular domain (anti-P2X7R-ec). (A,C): control samples with appropriate secondary antibodies. (E–H): differential expression of P2X7R isoforms. (E,G): staining with anti-P2X7R-Cter. (F,H): staining with anti-P2XR7-ec. (E,F): representative osteosarcoma stained with both antibodies (P2X7RA+B positive). (G,H): representative osteosarcoma stained only with anti-P2X7Rec (P2X7RB only positive). (I): number of cells per microscopic (40x) field in P2X7RA+B positive (purple) or P2X7RB only positive (cyan) tumour specimens. Cells were counted as described in Materials and Methods. Data are shown as means ± SE (N = 8, ***p<0.001).

**Figure 2:**
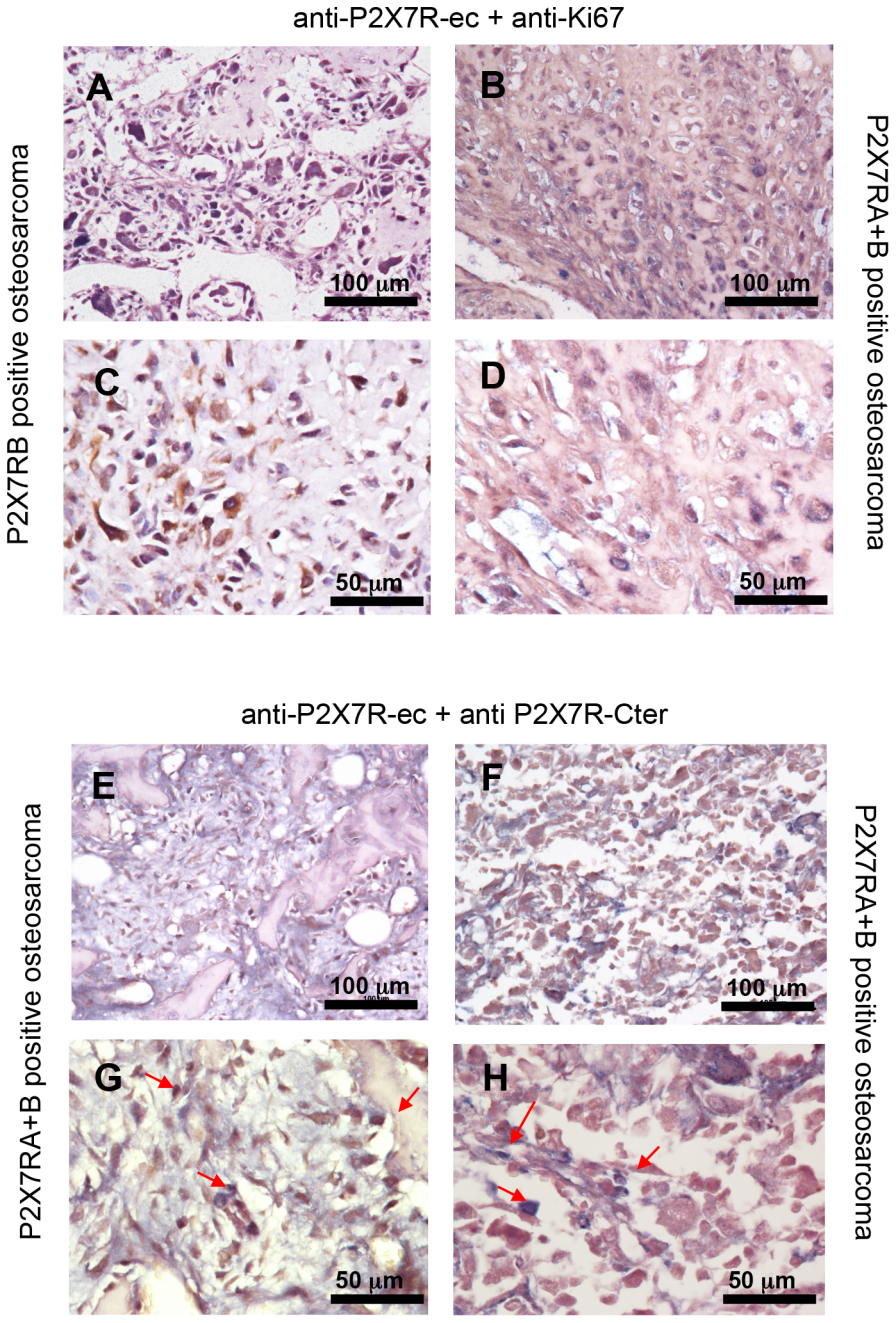
Double immunohistochemistry on human osteosarcomas. Co-immunostainings of osteosarcoma tissue array either with the anti-P2X7R-ec plus anti-Ki67 antibodies (A–D), or with the two primary anti-P2X7R antibodies (E–H) were carried out as described in Materials and Methods. (A,C): representative P2X7RB positive osteosarcoma showing a higher number of Ki67 positive nuclei (stained in blue) respect to a representative P2X7RA+B tumour (B,D). (E–H): representative P2X7RA+B positive tumours demonstrating the presence of a mix of cells some (stained in brown) positive for P2X7RB only, some others (stained in blue-brown) positive for both P2X7RA and B. Two different magnifications, 20x (A,B,E,F) and 40x (C,D,G,H) are shown.

A tissue array containing samples from 54 stage IV osteosarcomas was screened for P2X7RA and B expression by immunohistochemistry. Specimens included mainly osteoblastic and chondroblastic osteosarcomas from 39 male and 15 female patients, in the 5 to 61 age range. Two different antibodies were employed. A P2X7RA-selective polyclonal Ab, directed against the P2X7RA-specific C-terminal tail, here abbreviated as anti-P2X7R-Cter, and a monoclonal antibody recognizing both P2X7RA and B since raised against the extracellular domain common to both isoforms, here shortened as anti-P2X7R-ec. Antibodies reactivity was firstly checked on specimens from tumours obtained following inoculation of HEK293-mock and HEK293-P2X7RA cells into nude mice (not shown). Representative osteosarcoma samples stained by the two different antibodies are presented in Figure 1 (panels A-D). Of the osteosarcomas analysed, the great majority (44/54) (81.4%) stained positive with the anti-P2X7R-ec mAb, while 31/54 (57.4%) resulted positive with the anti-P2X7R-Cter Ab. Twenty nine samples (53.7%) resulted P2X7RA+B positive since labelled by both the anti-P2X7R-Cter Ab as and the anti-P2X7R-ec mAb (Figure 1E), whereas 15/54 (27.7%) resulted positive only for P2X7RB as staining was exclusively obtained using the anti-P2X7R-ec mAb, but not the anti-P2X7R-Cter Ab (Figure 1G,H). Evaluation of the number of cells per microscopic field demonstrated that osteosarcomas expressing uniquely P2X7RB were characterized by a higher cell density than osteosarcomas positive for both P2X7RA and B (Figure 1I). This was also confirmed by evaluation at double immunostaining of Ki67 labelled nuclei (Figure 2A–D). The number of Ki67 positive nuclei was higher in osteosarcomas positive uniquely for P2X7RB than in P2X7RA+B positive tumours (373.3±31.9 vs 189.3±40.9). Co-immunostaining of P2X7RA+B tumours with the two anti-P2X7R antibodies (Figure 2E–H) showed the presence of a mix of cells among which some were positive for P2X7RB only, whereas others resulted labelled for both isoforms (arrows in Figure 2G,H). Overall these results show that P2X7R is expressed in osteosarcomas and point out a link between P2X7RB expression and increased cell density.

### Expression and functional characterization of P2X7R in Te85 osteosarcoma cells

**Figure 3:**
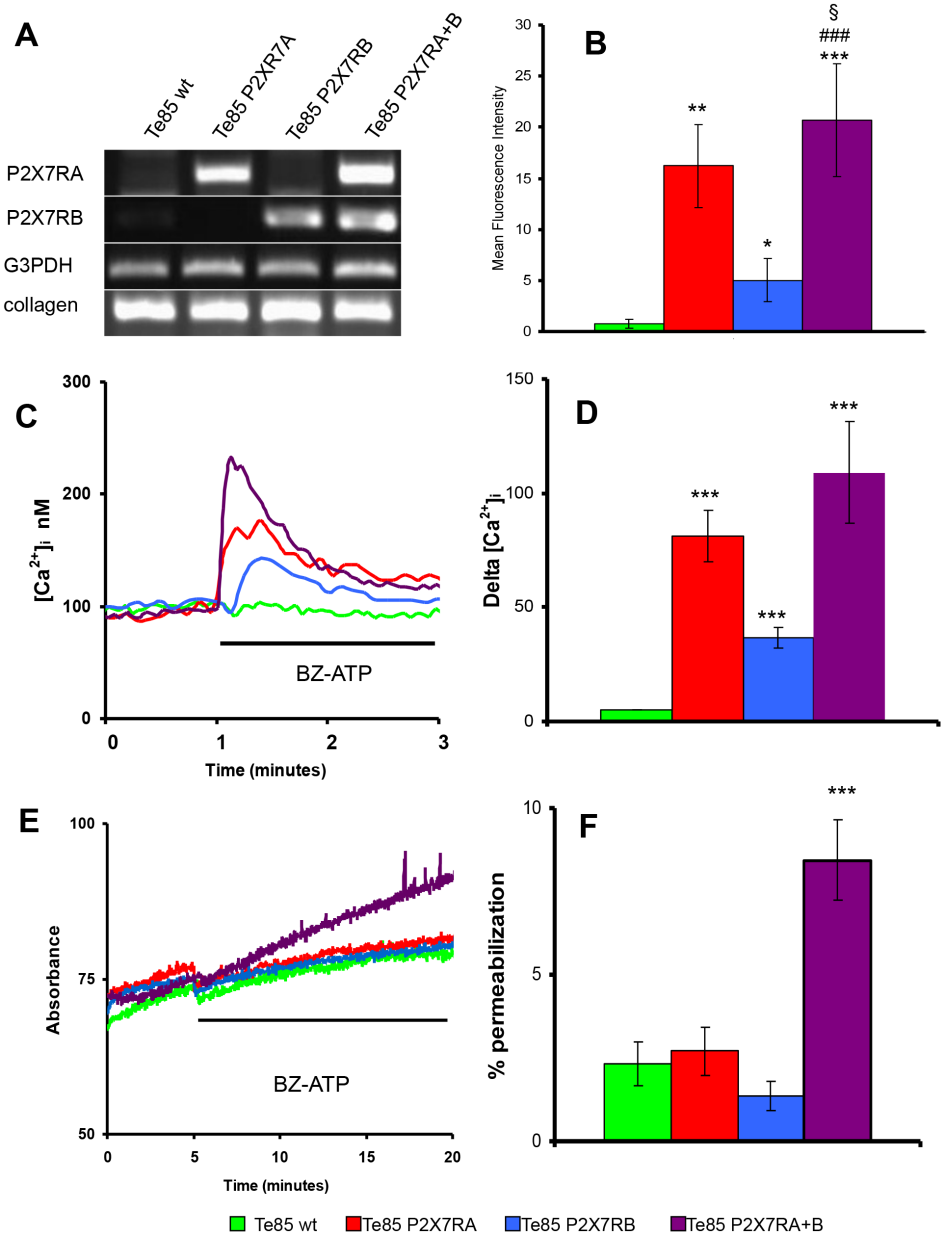
Characterization of P2X7R isoforms A and B expressed in Te85 transfected clones. (A): RT-PCR showing selective expression of P2X7RA or P2X7RB in Te85 clones. Housekeeping control gene was G3PDH, osteoblast specific gene was collagen I. (B): P2X7R surface expression determined by flow cytometry with anti-P2X7R-ec. Graph shows the Mean Fluorescence Intensity (MFI) after subtraction of secondary antibody values. Data are shown as means ± SE, N = 3. ***P<0.001 versus Te85 wt; ^###^ *P*<0.001 versus Te85 P2RX7B; §*P*<0.05 versus Te85 P2RX7A. (C): Representative traces showing intracellular calcium increment following stimulation with 500 µM BzATP. (D): Calcium variations evoked by P2X7R activation showed as means ± SE, N = 10. ***p<0.001 versus Te85 wt. (C): Representative traces showing ethidium bromide uptake following stimulation with 500 µM BzATP. (D): Percentages of ethidium permeabilization on digitonin control showed as means ± SE, N = 10. ***p<0.001 versus Te85 wt. Colour coding: green Te85 wt, red Te85 P2X7RA, cyan Te85 P2X7RB, purple Te85 P2X7RA+B.

High P2X7R expression by human osteosarcomas makes it difficult to investigate the effect of receptor overexpression in osteosarcoma cells. We thus used as a model the Te85 human osteosarcoma cell line which lacks endogenous P2X7R protein expression. Te85 cells were transfected with P2X7RA, P2X7RB or co-transfected with both (P2X7RA+B). Receptor expression was assessed by both RT-PCR and flow cytometry (Figure 3). RT-PCR showed a 400 bp band, corresponding to P2X7RA amplicon, in Te85-P2X7RA and in Te85-P2X7RA+B cells, while a 500 bp band, corresponding to P2X7RB, was detected in Te85-P2X7RB and Te85-P2X7RA+B cells (Figure 3A). Te85-wt cells and all P2X7R-transfected cells expressed collagen I as a marker of osteoblast differentiation (Figure 3A). Flow cytometry analysis, performed using the anti-P2X7R-ec mAb, showed that plasma membrane P2X7RA expression was higher than P2X7RB, and that the highest level of cell surface expression was achieved in Te85 cells transfected with both P2X7RA and P2X7RB (*p*<0.05 for P2X7RA+B versus P2X7RA) (Figure 3B). Stimulation with 500 µM BzATP (corresponding to the EC_50_ for P2X7RB which has a lower affinity for ATP than the full length P2X7RA [13]) triggered a [Ca^2+^]_i_ rise in all transfected clones (Figure 3C). Ca^2+^ increments occurred in the following order: Te85-P2X7RB < Te85-P2X7RA < Te85-P2X7RA+B (Figure 3D). This response might depend on the different plasma membrane expression level of the diverse isoforms (P2X7RB the lowest, P2X7RA+B the highest), or on the activation of the receptor-associated large conductance pore. To clarify this issue we tested pore formation in Te85 transfectants. As shown in Figure 3E, F, P2X7RB did not support any plasma membrane permeabilization, a result expected due to lack of the extended cytoplasmic C-tail associated to pore opening [13]. Unexpectedly, this was also the case for P2X7RA, which usually gates the large conductance pore. Interestingly, pore-forming activity was restored in Te85 cells transfected with both P2X7R variants (Figure 3E, F). To sum up, in order to reproduce the typical P2X7R signature in Te85 osteosarcoma cells, expression of both P2X7RA and B isoforms is clearly needed.

### P2X7R transfection triggers extracellular ATP release, NFATc1 activation, and drives proliferation of Te85 osteosarcoma cells

**Figure 4:**
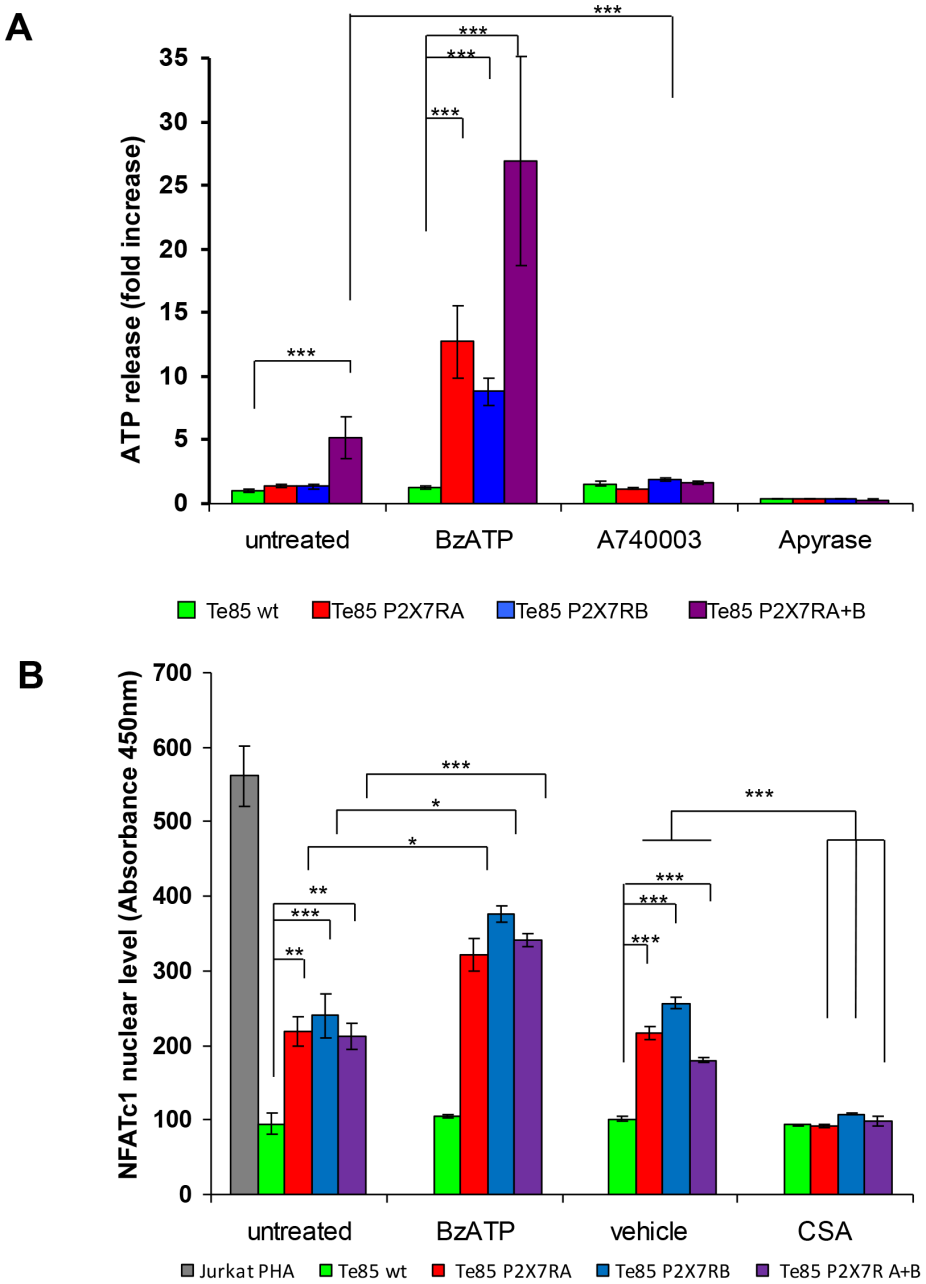
P2X7RA and B increase ATP release and NFATc1 activity in Te85 transfected cells. (A): Extracellular ATP was measured in the culture supernatants with ENLITEN rLuciferase/Luciferin reagents as described in materials and methods. Te85 clones were plated at 5x10^5^ cells per well in 96 well plates and, following adhesion, incubated for 24 hours in the absence (untreated) or presence of either 100 µM BzATP, 100 µM A740003 or 4 U/ml Apyrase. ATP release was expressed as fold increase over Te85 wt reference sample. Data are means ± SE; N = 3. (B) Nuclear fractions of the different clones were obtained as described in materials and methods. Activated NFATc1 was measured by ELISA in absence (untreated) or in presence of either 50 µM BzATP or 10 µM cyclosporin (CSA). CSA vehicle (DMSO) was used as control. Results are compared to nuclear NFATc1 levels in Jurkat cells stimulated with PHA supplied by manufacturer. In graph means ± SE of absorbance are shown, N = 9. Colour coding: green Te85 wt, red Te85 P2RX7A, cyan Te85 P2RX7B, purple Te85 P2RX7A+B.

Extracellular ATP release from Te85 wt and transfected cells was measured. Among all cell lines tested, only Te85 cells transfected with both P2X7RA and B showed a significantly higher value than Te85 wt cells (Figure 4A). Therefore, pore formation found in Te85 P2X7RA+B cells appears related to extracellular ATP release. Extracellular ATP was significantly increased in all transfectants by BzATP treatment, reduced by the selective P2X7R antagonist A740003, and fully abrogated upon administration of the ATP degrading-enzyme, apyrase (Figure 4A).

Intracellular calcium mobilization is one of the main stimuli leading to activation of the nuclear factor of activated T cells complex 1 (NFATc1) which has been associated to P2X7R-dependent proliferation [11] and is also known to be central in osteoblast biology [43]. Analysis of NFATc1 nuclear translocation showed that all P2X7R-transfected Te85 clones had significantly higher nuclear NFATc1 levels than Te85 wt cells, although no major differences were observed among the transfected clones (Figure 4B). BzATP treatment significantly increased the levels of activated NFATc1 in all clones whereas cyclosporin A (CSA), a well known NFATc1 inhibitor, took the NFATc1 activation levels back to that of Te85 wt control cells (Figure 4B).

**Figure 5:**
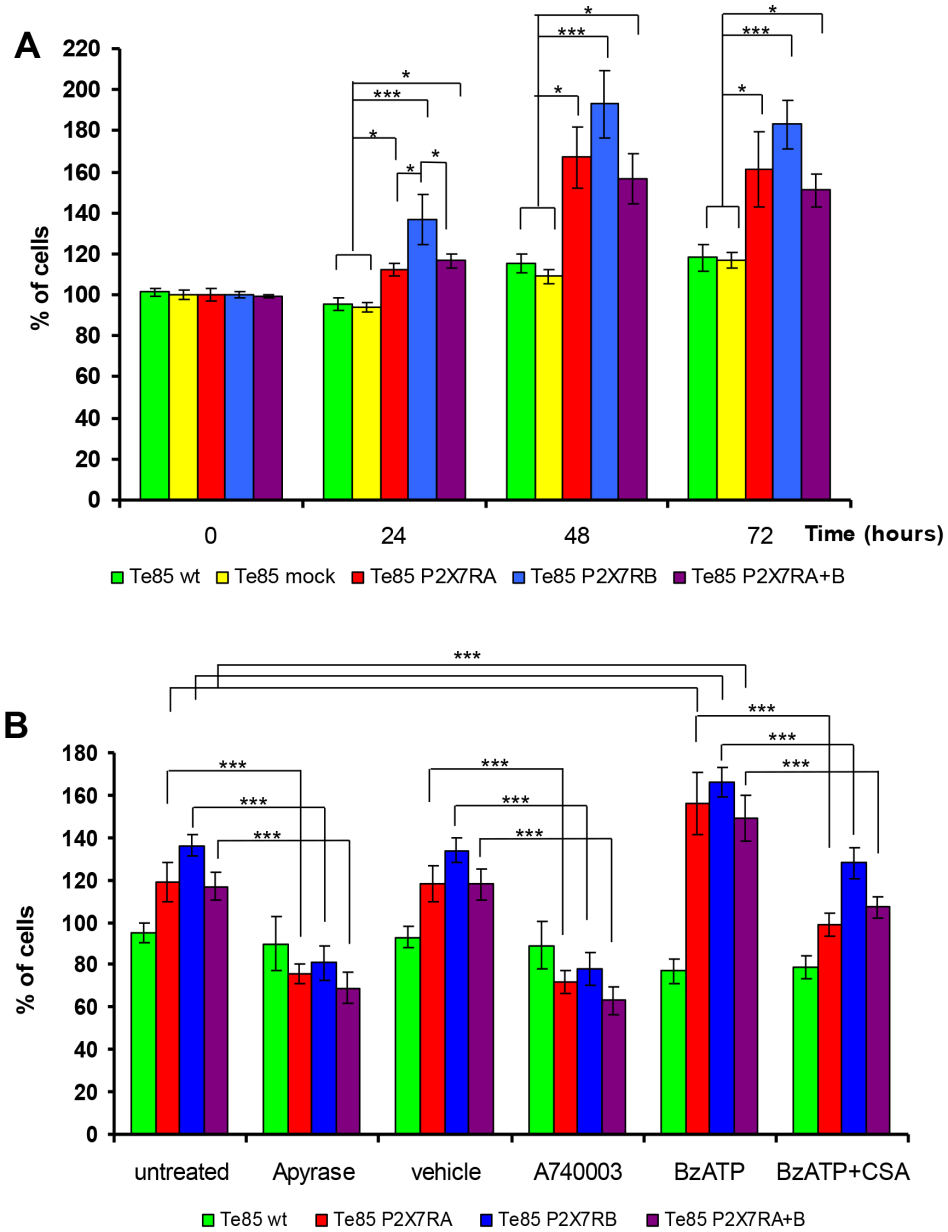
P2X7RA and B increase cell growth in Te85 transfected cells. (A): Te 85 clones were plated at a 10^5^ cells/ml in 6 well plates in cell culture medium without FBS. Percentage of viable cells was evaluated at the different time points as described in materials and methods. The graph shows means ± SE, N = 12. *p<0.05, ***P<0.001 versus Te85 wt control; ^#^ *p*<0.05 versus Te85 P2RX7A+B, ^$^ *p*<0.05 versus Te85 P2RX7A. (B) Cell proliferation assessed for 24 hours in absence (untreated) or presence of: 4 U/ml Apyrase, 100 µM A740003, 100 µM BzATP, 100 µM BzATP +10 µM cyclosporin (CSA). A740003 and CSA vehicle (DMSO) was used as control. The graph shows means ± SE, N = 3. Colour coding: green Te85 wt, yellow Te85mock, red Te85 P2RX7A, cyan Te85 P2RX7B, purple Te85 P2RX7A+B.

Converging evidence from different laboratories show that both P2X7RA and B are endowed with a strong growth-promoting activity [10], [13], [44], [45], and in particular P2X7RA is overexpressed in many human malignant tumours [46]–[49]. To check whether P2X7R might also support osteosarcoma cell growth, we investigated the effect of P2X7R isoforms on Te85 cell proliferation. P2X7R expression was indeed a powerful stimulus for cell growth, the most efficient growth-promoting isoform being P2X7RB (Figure 5A). Treatment with either apyrase or A740003, significantly reduced, whereas BzATP stimulation significantly increased, the proliferative capability of all transfectants (Figure 5B). This suggests the existence of an ATP-mediated loop controlling and sustaining cell proliferation. To confirm the central role played by NFATc1 in P2X7R-sustained proliferation, the effect of CSA was tested on Te85 cell growth. As shown in Figure 5B, CSA reduced BzATP-induced proliferation of all transfectants proving the reliance of P2X7R-dependent proliferation on NFATc1 activation in our model.

### P2X7R expression modulates RANK-L, OPG and mineralization

**Figure 6:**
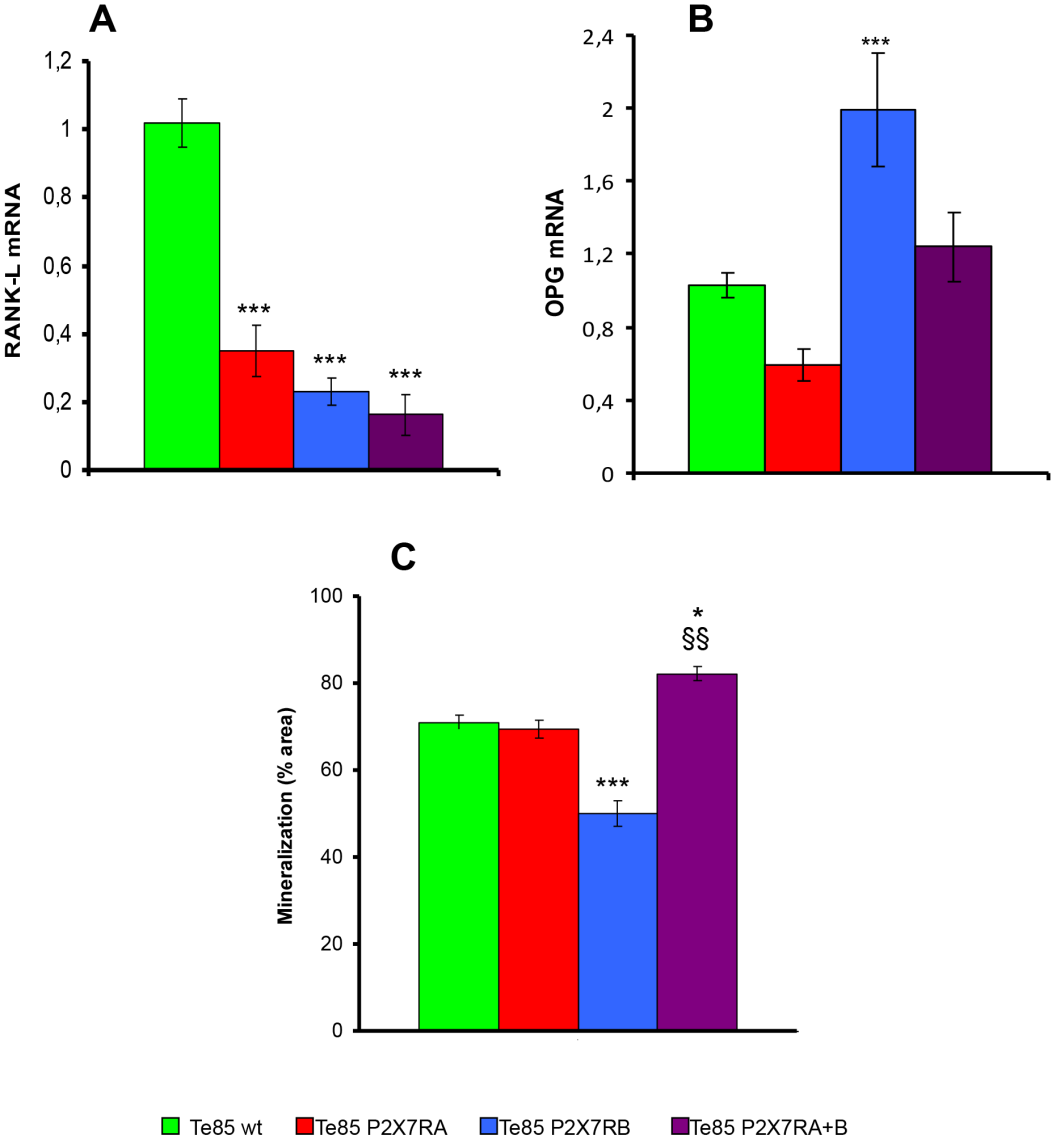
P2X7RA and B expression reduces RANK-L while differently modulates OPG messenger and mineralization. RANK-L and OPG mRNA levels were evaluated by real-time PCR as described in Materials and Methods. Messenger expression was normalized on G3PDH internal control and displayed as fold increase over Te85 wt reference sample. (A): RANK-L mRNA expression (B): OPG mRNA expression. Data are shown as mean ± SE, N = 12, ***p<0.001 versus Te85 wt. (C): Mineralization by Te85 clones was assessed over a 21 days period as described in Materials and Methods. (A): Quantification of the amount of alizarin red S staining of each clone. Data are shown as means ± SE (N = 6) analysed by Kruskal-Wallace test with Dunn's multiple comparison post-test. ***P<0.001 versus Te85 wt and Te85-P2X7RA; *P<0.05 vs Te85 wt; ^$$^ *P*<0.01 versus Te85 P2X7RA. Colour coding: green Te85 wt, red Te85 P2X7RA, cyan Te85 P2X7RB, purple Te85 P2X7RA+B.

Function of P2X7R on osteosarcoma biology was further investigated by checking the expression of two crucial molecules for bone homeostasis, i.e. receptor activator of nuclear factor kappa B-ligand (RANK-L) and osteoprotegerin (OPG). While RANK-L messenger was reduced in all P2X7R-expressing clones (Figure 6A), OPG mRNA was significantly increased only in P2X7RB transfected cells (Figure 6B). However, the RANK-L/OPG ratio was decreased in all P2X7R clones. A reduced RANK-L/OPG ratio is generally associated *in vivo* with decreased bone resorption, a powerful boost for bone mass and a feature of osteosclerotic lesions. Finally, the effect of P2X7R expression on mineralization, another relevant osteoblastic activity, was assessed. In this case, the transfection with the two isoforms had quite different effects (Figure 6C). P2X7RA alone did not significantly modify mineralisation as compared to Te85 wt cells. On the contrary, expression of P2X7RB caused a striking reduction of mineralisation respect to Te85 wt and Te85-P2X7RA, whereas a significant increase was observed in cells transfected with both P2X7RA and B variants (Figure 6C).

## Discussion

Interaction of tumour cells with ligand molecules present in the tumour microenvironment is central in cancer growth and progression. ATP recently emerged as an extracellular messenger present at high levels in the tumour microenvironment [50]–[52], but the effect of this molecule on carcinogenesis is yet incompletely known. In a recent paper we demonstrated that P2X7R is involved in tumour growth and neo-vascularization *in vivo* [5]. These data corroborated previous results showing that the P2X7R supports proliferation of lymphocytes [9], osteoblasts [16] and osteosarcoma cells [53].

In the present study, we report that human osteosarcomas express at high level the full length P2X7RA and the truncated P2X7RB isoforms, two variants previously shown to support cell proliferation [13]. Staining with two different antibodies recognizing either both isoforms or P2X7RA only, allows us to conclude that most osteosarcomas analysed express both P2X7RA and P2X7RB. The possibility that isoforms different from P2X7RA or P2X7RB are recognized by the anti-P2X7R-ec antibody is very unlikely as all other splice variants (P2X7RC-J) either loose or gain introns in the extracellular domain recognized by the antibody [41]. On the other hand, if expressed, non-functional P2X7RC, P2X7RD, P2X7RF and P2X7RH isoforms would be recognized by anti-P2X7R-Cter as they carry the same C-terminal tail as P2X7RA. However, positivity of 27.7% of total osteosarcomas just for the anti-P2X7R-ec strongly suggests that the only variant expressed by these tumours is P2X7RB. Furthermore, the finding that P2X7RB positive osteosarcomas showed higher cell density and increased Ki67 positivity than those expressing both isoforms indicates a relationship between P2X7RB expression and enhanced cell proliferation.

P2X7R might sustain osteosarcoma growth in an autonomous fashion thanks to autocrine/paracrine ATP release. Further, it might also modulate osteosarcoma cell interaction with other bone cells by regulating release of key molecules such as RANK-L and OPG or by affecting osteodeposition. To deeply investigate these points, Te85 osteosarcoma cells, lacking endogenous P2X7R protein expression, were stably transfected with either P2X7RA, P2X7RB or both. Our data show that all P2X7R-transfected Te85 osteosarcoma clones displayed increased proliferation, compared to Te85 wt or Te85 mock cells, thus confirming a trophic activity of P2X7R also in this tumour. Receptor stimulation by BzATP significantly increased proliferative activity of all transfected clones, Te85 P2X7RB cells presenting the highest growth ability. The latter result is in agreement with the immunostaining of osteosarcomas in which the truncated P2X7RB isoform was present and related to higher cell number. Central to P2X7R mediated proliferation is NFATc1 activation. This nuclear factor, besides being one of the recognized mediators of P2X7R dependent growth [11], is also a known determinant of osteoblast proliferation [43] and bone formation [54]. NFATc1 activity was indeed increased in all P2X7R transfectants, improved by BzATP and decreased by CSA treatment.

Interestingly, P2X7RA and P2X7RB isoforms have differential effects on cell growth and on mineralization. In fact, P2X7RB, which is unable to generate the large conductance pore, confers the highest growth drive, while strongly reduces osteodeposition. On the contrary, P2X7RA causes a smaller increase in cell growth, whereas mineralization is not significantly different from that observed in the Te85 wt cells. Finally, co-transfection with both isoforms causes a cell growth increase comparable to that of P2X7RA alone but a stronger stimulus for mineralization.

**Figure 7:**
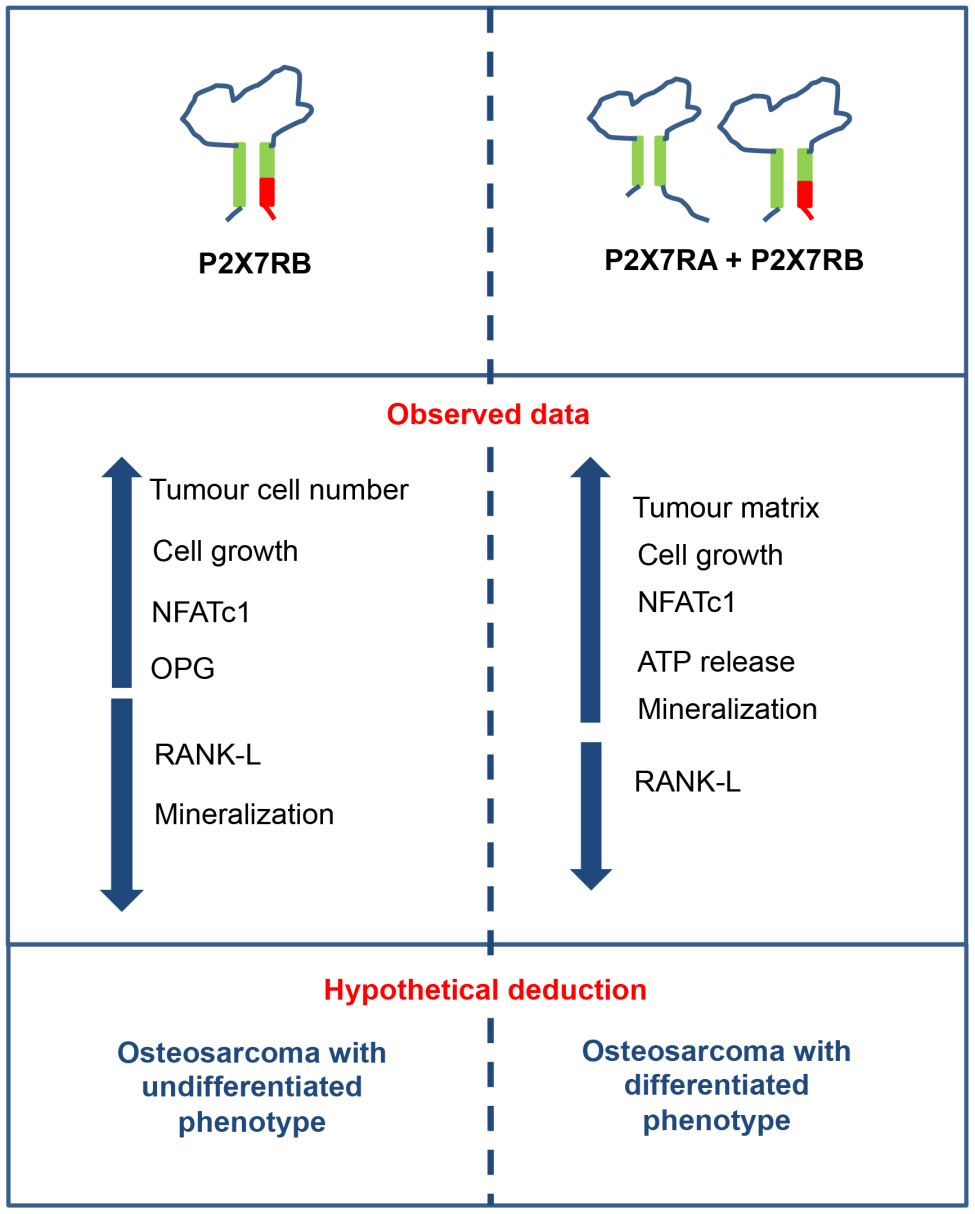
Schematic summary of obtained data and hypothetical deductions.

Previous studies reported atypical P2X7R pharmacology in osteosarcoma cell lines [22]. Accordingly, our data show that, in order to produce P2X7R full-blown physiological signature, i.e. formation of both channel and large conductance pore, expression of P2X7RA in Te85 cells is not sufficient but co-expression of the shorter variant (P2X7RB) is also required. In fact, only Te85 P2XR7A+B cells undergo a small but significant permeabilization to high molecular weight solutes, possibly due to either increased cell surface expression, as detected at flow cytometry, or interaction with a different subset of intracellular proteins [55]. P2X7RA+B dependent pore forming activity was accompanied by increased spontaneous ATP release that could possibly enhance mineralization [13], [40], [56]. Increased proliferation and reduced bone deposition characterizing Te85 P2X7RB cells might be suggestive of a de-differentiated phenotype. This is also indicated by the reduced extracellular matrix in osteosarcomas positive at immunostaining for P2X7RB compared to that of tumours positive for both P2X7RA and P2X7RB. In summary, the opposite effects of P2X7RB and P2X7RA+B on osteosarcoma cell growth and mineralization (Figure 7), suggests that P2X7R as an ion channel is predominantly involved in cell proliferation, while activation of the P2X7R-associated large conductance pore might be chiefly responsible for the differentiation-associated effects.

A role for P2X7R in bone formation is supported by the findings of Panupinthu et al. [16] who found that osteoblasts isolated from P2X7R-deficient mice showed reduced bone deposition. Contrary to these findings, other *in vitro* reports suggest that ATP spontaneously released from rat osteoblasts and acting at P2X7R was responsible for reduced bone formation [57]. In our experimental model, P2X7R-mediated ATP release seems related to augmented mineralizing activity, as shown by P2X7RA+B expressing cells behaviour. Additionally, our study points out differences between the two isoforms, in particular showing a relation between P2X7RB expression and proliferation, likely at the expense of bone mineralization. The small number of human osteosarcomas analysed and limited clinical data in our possession do not allow us to conclude whether osteosarcomas expressing P2X7RB behave more aggressively. Analysis of a larger patient sample will help in clarifying this issue.

P2X7R transfection also affects expression of RANK-L and OPG, two crucial molecules in bone remodelling. Osteosarcomas generally exhibit altered bone remodelling due to either excessive deposition or osteolysis. Our data show that P2X7R expression significantly reduces RANK-L messenger levels in all transfectants. Instead, OPG levels were increased only in Te85 P2X7RB. Nevertheless, RANK-L/OPG ratio was reduced in all P2X7R expressing clones. A low RANK-L/OPG ratio on one hand will be beneficial for osteosarcoma patients, as it decreases osteolysis and related pain [58], whereas, on the other, it will be negative as it increases invasiveness [4], [59].

In conclusion, our study demonstrates expression and trophic activity of P2X7R receptor isoforms A and B in human osteosarcoma. These data support a role of P2X7R in osteosarcoma growth and bone remodelling and point to P2X7 receptor as a potential target for osteosarcoma therapy.
